# Supplementary material for: Resolving the full spectrum of human genome variation using Linked-Reads
Source: Genome Res. 2019 Apr;29(4):635–45. doi: 10.1101/gr.234443.118 (PMC6442396; doi:10.1101/gr.234443.118)
Supplement: Supplemental Material [file supp_gr.234443.118_Supplemental_longranger-2.2.zip › longranger-2.2.tar.bz2!/lr2.2-public/lib/stan/cmdstan/stan/doc/api/html/agrad_2fwd_2functions_2log__rising__factorial_8hpp_source.html]

Stan: src/stan/agrad/fwd/functions/log\_rising\_factorial.hpp Source File


|  |  |
| --- | --- |
|  | Stan  2.5.0  probability, sampling & optimization |


- Main Page
- Related Pages
- Namespaces
- Classes
- Files

- File List
- File Members

All Classes Namespaces Files Functions Variables Typedefs Enumerations Enumerator Friends Macros Pages

- src
- stan
- agrad
- fwd
- functions

log\_rising\_factorial.hpp

Go to the documentation of this file.

1 #ifndef STAN\_\_AGRAD\_\_FWD\_\_FUNCTIONS\_\_LOG\_RISING\_FACTORIAL\_HPP

2 #define STAN\_\_AGRAD\_\_FWD\_\_FUNCTIONS\_\_LOG\_RISING\_FACTORIAL\_HPP

3

4 #include <stan/agrad/fwd/fvar.hpp>

5 #include <stan/meta/traits.hpp>

6 #include <stan/math/functions/log\_rising\_factorial.hpp>

7 #include <boost/math/special\_functions/digamma.hpp>

8

9 namespace stan {

10

11  namespace agrad {

12

13  template<typename T>

14  inline

15  fvar<T>

16  log\_rising\_factorial(const fvar<T>& x, const fvar<T>& n) {

17  using stan::math::log\_rising\_factorial;

18  using boost::math::digamma;

19

20  return fvar<T>(log\_rising\_factorial(x.val\_,n.val\_),

21  (digamma(x.val\_ + n.val\_) \* (x.d\_ + n.d\_) - digamma(x.val\_) \* x.d\_));

22  }

23

24  template<typename T>

25  inline

26  fvar<T>

27  log\_rising\_factorial(const fvar<T>& x, const double n) {

28  using stan::math::log\_rising\_factorial;

29  using boost::math::digamma;

30

31  return fvar<T>(log\_rising\_factorial(x.val\_,n),

32  (digamma(x.val\_ + n) - digamma(x.val\_)) \* x.d\_);

33

34  }

35

36  template<typename T>

37  inline

38  fvar<T>

39  log\_rising\_factorial(const double x, const fvar<T>& n) {

40  using stan::math::log\_rising\_factorial;

41  using boost::math::digamma;

42

43  return fvar<T>(log\_rising\_factorial(x,n.val\_),

44  (digamma(x + n.val\_) \* n.d\_));

45  }

46  }

47 }

48 #endif

log\_rising\_factorial.hpp

stan::agrad::fvar

**Definition:** fvar.hpp:13

traits.hpp

stan::math::log\_rising\_factorial

boost::math::tools::promote\_args< T1, T2 >::type log\_rising\_factorial(const T1 x, const T2 n)

**Definition:** log\_rising\_factorial.hpp:41

fvar.hpp

stan::agrad::digamma

fvar< T > digamma(const fvar< T > &x)

**Definition:** digamma.hpp:16

stan::agrad::fvar::d\_

T d\_

**Definition:** fvar.hpp:16

stan::agrad::log\_rising\_factorial

fvar< T > log\_rising\_factorial(const fvar< T > &x, const fvar< T > &n)

**Definition:** log\_rising\_factorial.hpp:16

stan::agrad::fvar::val\_

T val\_

**Definition:** fvar.hpp:15


---

[ Stan Home Page ]

*© 2011–2014,
Stan Development Team.*
